# Distinct and shared gene expression for human innate versus adaptive helper lymphoid cells

**DOI:** 10.1002/JLB.5MA0120-209R

**Published:** 2020-02-04

**Authors:** Giuseppe Ercolano, Tania Wyss, Bérengère Salomé, Pedro Romero, Sara Trabanelli, Camilla Jandus

**Affiliations:** ^1^ Department of Oncology UNIL CHUV and Ludwig Institute for Cancer Research Lausanne University of Lausanne Lausanne Switzerland; ^2^ Department of Oncology UNIL CHUV University of Lausanne Lausanne Switzerland

**Keywords:** CD4 T helper cells, human, ILCs, lncRNAs, RNAseq

## Abstract

Innate lymphoid cells (ILCs) are the latest identified innate immune cell family. Given their similarity in transcription factor expression and cytokine secretion profiles, ILCs have been considered as the innate phenocopy of CD4 Th cells. Here, we explored the transcriptome of circulating human ILC subsets as opposed to CD4 Th cell subsets. We describe transcriptomic differences between total ILCs and total CD4 Th cells, as well as between paired innate and adaptive cell subsets (ILC1 vs. Th1; ILC2 vs. Th2; and ILC3 vs. Th17 cells). In particular, we observed differences in expression of genes involved in cell trafficking such as CCR1, CCR6 and CXCR3, innate activation and inhibitory functions, including CD119, 2B4, TIGIT, and CTLA‐4, and neuropeptide receptors, such as VIPR2. Moreover, we report for the first time on distinct expression of long noncoding RNAs (lncRNAs) in innate vs. adaptive cells, arguing for a potential role of lncRNA in shaping human ILC biology. Altogether, our results point for unique, rather than redundant gene organization in ILCs compared to CD4 Th cells, in regard to kinetics, fine‐tuning and spatial organization of the immune response.

## Graphical abstract

Human ILCs and CD4 Th cells show shared, but also distinct transcriptomic profiles, including in lncRNAs, supporting their distinct temporal and spatial activation during immune responses.

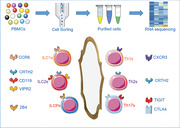

**Abbreviations**
ES: Enrichment score
GO: Gene ontology
GSEA: Gene set enrichment analysis
ILCs: Innate lymphoid cells
MCMV: Murine cytomegalovirus
NES: Normalized enrichment score
PCA: Principal component analysis
PCs: Principal components
PRC2: Polycomb repressive complex 2
Th cells: T helper cells
TMM: Trimmed mean of M value
Tregs: Regulatory T cells

## 1 INTRODUCTION

Innate lymphoid cells (ILCs) have recently emerged as a key contributors to host defense and tissue homeostasis, given their capacity to rapidly respond to microenvironmental cues.^1^ ILCs are a family of lymphoid cells that, in contrast to adaptive T and B lymphocytes, do not express receptors for antigens. Similar to T cells, ILCs exhibit functional specialization with the ability to produce cytokine in patterns resembling helper CD4 T cells. Helper ILCs are subdivided into 3 main subsets, ILC1s, ILC2s, and ILC3s, according to their master transcription factor expression and cytokine production profiles. ILC1s express Tbet and mainly secrete IFNγ during infections by intracellular pathogens, ILC2s express GATA3 and secrete IL‐5 and IL‐13 in response to helminths and ILC3s express RORγt and secrete IL‐17 in the context of infections by extracellular microorganisms. However, whereas this categorization holds true in tissues, ILC3s are under‐represented in the circulating lymphocyte pool and included in a population of ILC precursors able to give rise to all ILC subsets. Therefore, circulating c‐Kit^+^CRTH2^−^ ILCs are now referred to ILCPs.^2^ Overall, ILCs have been often considered as innate redundant mirrors of CD4 Th cells, ILC1s being the innate counterpart of Th1s, ILC2s of Th2s and ILC3s/ILCPs of Th17s. Whether this apparent functional equivalency holds true at higher levels of resolution both cellularly and molecularly remains unexplored.

Several bulk and single‐cell transcriptomic analyses have been performed in both mouse and human ILCs. In mice, these studies allowed to clarify the developmental positioning of each ILC subset, but also to appreciate the impact of the microenvironment, including the microbiota, in shaping their transcriptional identity. By comparing the gene expression of ILCs and NK cells from different organs, it was shown that unique profiles are acquired by ILC subsets, whereas overlaps in gene expression are present among ILC1s and NK cells.^3^, ^4^ In another study, the authors showed a functional sub‐compartmentalization within the main ILC subsets, mainly within ILC3s, in tissues such as the intestinal mucosa.^5^ These properties might be key for ILCs to rapidly integrate and adapt to environmental stimuli. In humans, a single‐cell mRNAseq analysis of tonsil derived ILCs also revealed distinct transcriptional programs in each ILC population and functional sub‐specialization within ILC subsets, particularly in tonsil‐derived ILC3s.^6^ Yudanin and colleagues have provided a transcriptomic mapping of ILCs in nondiseased human tissues, highlighting spatial and temporal characteristics of individual ILC subsets.^7^ Last, a recent gene expression profiling of human circulating ILCs enabled to identify unique ILC signatures of each individual ILC subset.^8^

These advances in our understanding of ILC biology argues for complementary, but also divergent functions of ILCs and CD4 Th cells in host immune responses. In fact, despite the presence of the exact same master transcription factors in mirrored ILC and CD4 Th subsets, functional distinction cannot be excluded. Evidence in that direction comes from studies on ILC3 activity using RAG knockout and wild‐type mice where antagonist interactions have been described for gut resident ILC3s and Th17 cells.^9^, ^10^ In line with these findings, a distinct temporal activity for ILC3s and their adaptive counterparts was also reported in the context of *Citrobacter rodentium* infections. In that setting, sequential ILC and Th functions were shown to be necessary for pathogen clearance, the action of T cells contributing to extinguish the early functions of ILC3.^12^, ^13^ Further, parallel gene expression profiling and epigenetic analysis of murine ILC and CD4 Th subsets at steady state revealed shared, but also different networks of functional regulators between innate and adaptive cytokine secreting cells, as well as among subsets within the same lineage. Interestingly, regulatory circuits in both lineages are dramatically altered in the context of Type 2 infection models, but at the same time converge to a similar epigenetic signature. Strikingly, ILC regulomes appear to be already poised before cell activation, possibly explaining the ability of ILCs to rapidly respond to infections. In contrast, CD4 Th regulatory elements undergo considerable remodeling during antigen stimulation. Whereas these comparisons have allowed us to revise our view on ILC‐CD4 Th cell analogies in model organisms, knowledge about the transcriptomic similarities between human ILCs and CD4 Th cells is still limited. In a study, regulomes of human tonsil‐derived ILC1s and ILC3s were compared to the ones of Th1 and Th17 cells, respectively, showing the presence of both unique and overlapping pathways in innate and adaptive mirror cells.^3^ However, due to the paucity of ILC2s and Th2s in tonsils, the investigation of these cells was not included in that analysis. Furthermore, no data are available on the comparison of ILCs and CD4 Th cells in the human peripheral blood.

In the current study, we compared gene expression profiles of human circulating helper ILCs and CD4 Th cells. We show transcriptomic differences in expression of genes involved in cell trafficking, innate activation, and inhibitory functions, supporting distinct temporal and spatial activation of ILCs and Th cells in vivo. Moreover, we report on distinct expression of long noncoding RNAs (lncRNAs) in innate vs. adaptive cells, arguing for a subtle and different cellular fine‐tuning of human ILCs as compared to their adaptive counterparts.

## 2 METHODS

### 2.1 Cell preparation

Buffy coats were obtained from healthy donors at the local Blood Transfusion Center, Lausanne, Switzerland. PBMCs were isolated by density‐gradient centrifugation and immediately used.

### 2.2 Flow cytometry analysis and cell sorting

Isolated PBMCs were stained in sorting buffer (PBS, 50 μM EDTA, 0.2% BSA) with the following specific lineage marker Abs: anti‐human CD8 (MEM‐ 31, Immunotools, Friesoythe, Germany), anti‐human CD14 (RMO52, BC, Marseille, France), anti‐human CD15 (80H5, BC Marseille, France), anti‐human CD16 (3G8, BC, Marseille, France), anti‐human CD19 (J3‐119, BC, Marseille, France), anti‐human CD20 (2H7, Biolegend, San Diego, CA, USA), anti‐human CD33 (HIM3‐4, Biolegend, San Diego, CA, USA), anti‐human CD34 (561, Biolegend, San Diego, CA, USA), anti‐human CD203c (E‐NPP3) (NP4D6, Biolegend, San Diego, CA, USA), anti‐human FcεRIα (AER‐37, Biolegend, San Diego, CA, USA). Additionally, we used Brilliant Violet 421 anti‐human CD127 (IL‐7Rα) (A019D5, Biolegend, San Diego, CA, USA), Brilliant Violet 605 anti‐human CD3 (OKT3, Biolegend, San Diego, CA, USA), Alexa Fluor 700 anti‐human CD4 (RPA‐T4, Biolegend, San Diego, CA, USA), PerCPCy5.5 anti‐human CD56 (HCD56, Biolegend, San Diego, CA, USA), Brilliant Violet 785 anti‐human CD45RO (UCHL1, Biolegend, San Diego, CA, USA), PECy7 anti‐human CXCR3 (1C6, Biolegend, San Diego, CA, USA), APC/Fire 750 anti‐human CD117 (c‐kit) (104D2, Biolegend, San Diego, CA, USA), PE anti‐human CRTH2 (BM16, Biolegend, San Diego, CA, USA), APC anti‐human CD196 (CCR6) (G034E3, Biolegend, San Diego, CA, USA). In addition, cells were stained with the LIVE/DEAD Fixable Green Dead Cell Stain Kit (Life Technologies, Grand Island, NY, USA). Total ILCs were sorted as CD3^−^CD4^−^Lineage^−^CD127^+^, total CD4 Th cells as CD3^+^CD4^+^CD45RO^+^. ILC subsets for mRNA sequencing and quantitative real time PCR (qPCR) experiments were sorted based on the expression of cKit, CD56 and CRTH2 surface markers. More specifically, ILC1s were identified as CRTH2^−^cKit^−^CD56^−^, ILC2s as CRTH2^+^cKit^+/−^ and ILCPs as the cKit^+^CRTH2^−^. CD4 Th subsets for mRNA sequencing and qPCR experiments were sorted based on the expression of CXCR3, CRTH2, and CCR6. Th1s were identified as CXCR3^+^CRTH2^−^CCR6^−^, Th2s as CRTH2^+^CXCR3^−^, and Th17s as CXCR3^−^CRTH2^−^CCR6+. Cells were sorted using the FACSAria Fusion cell sorter (BD Bioscience, San Jose, CA, USA) or the MoFlo Astrios cell sorter (Beckamn Coulter, Marseille, France).

### 2.3 ILC and CD4 Th cell evaluation by flow cytometry

Human total ILCs and ILC subsets were identified using lineage markers, all FITC conjugated, that include: anti‐human CD3 (UCHT1, Beckman Coulter [BC], Marseille, France), anti‐human CD4 (SFCI12T4D11, BC, Marseille, France), anti‐human CD8 (MEM‐ 31, Immunotools, Friesoythe, Germany), anti‐human CD14 (RMO52, BC, Marseille, France), anti‐human CD15 (80H5, BC, Marseille, France), anti‐human CD16 (3G8, BC, Marseille, France), anti‐human CD19 (J3‐119, BC, Marseille, France), anti‐human CD20 (2H7, Biolegend, San Diego, CA, USA), anti‐human CD33 (HIM3‐4, Biolegend, San Diego, CA, USA), anti‐human CD34 (561, Biolegend, San Diego, CA, USA), anti‐human CD203c (E‐NPP3) (NP4D6, Biolegend, San Diego, CA, USA), anti‐human FcεRIα (AER‐37, Biolegend, San Diego, CA, USA). Additional markers used include: Brilliant Violet 421 anti‐human CD127 (IL‐7Rα) (A019D5, Biolegend, San Diego, CA, USA) Brilliant Violet 605 anti‐human CD117 (cKit) (104D2, Biolegend, San Diego, CA, USA), PerCPCy5.5 anti‐human CRTH2 (HCD56, Biolegend, San Diego, CA, USA), and BUV737 anti‐human CD56 (NCAM16.2, BD Horizon, San Jose, CA, USA). Human CD4 Th cells and Th cell subsets were identified using Brilliant Violet 605 anti‐human CD3 (OKT3, Biolegend, San Diego, CA, USA), BUV737 anti‐human CD4 (M5E2, BD Horizon, San Jose, CA, USA), FITC anti‐human CXCR3 (CD183) (G025H7, Biolegend, San Diego, CA, USA), PerCPCy5.5 anti‐human CRTH2 (BM16, Biolegend, San Diego, CA, USA), Brilliant Violet 785 anti‐human CD45RO (UCHL1, Biolegend, San Diego, CA, USA), Brilliant Violet 650 anti‐human CCR6 (CD196) (G034E3, Biolegend, San Diego, CA, USA), or Alexa Fluor 700 anti‐human CCR6 (CD193) (G034E3, Biolegend, San Diego, CA, USA). Dead cells were excluded using the LIVE/DEAD Fixable Green Dead Cell Stain Kit (Life Technologies, Grand Island, NY, USA). Additional markers were evaluated using PECy7 anti‐human CCR1 (5F10B29, Biolegend, San Diego, CA, USA), PE anti‐human lymphotoxin beta receptor (LTβR) (31G4D8, Biolegend, San Diego, CA, USA), PE/Dazzle 594 anti‐human CD152 (CTLA‐4) (BNI3, Biolegend, San Diego, CA, USA), PE/Dazzle 594 anti‐human TIGIT (A15153G, Biolegend, San Diego, CA, USA), PE anti‐human CD119 (IFN‐γ R α chain) (GIR‐44, Biolegend, San Diego, CA, USA), PE/Dazzle 594 anti‐human CD223 (LAG3) (11C3C65, Biolegend, San Diego, CA, USA), and APC/Cy7 anti‐human CD244 (2B4) (C1.7, Biolegend, San Diego, CA, USA). Samples were acquired on LSRFortessa (BD, San Jose, CA, USA) and data were analyzed using FlowJo software (TreeStar V.10).

### 2.4 mRNA extraction and sequencing library preparation

Pure FACS‐sorted cells were stored at −80°C in RNAlater (Thermo Fisher, Carlsbad, CA, USA), as previously reported.^11^ mRNA was extracted upon thawing using the RNeasy Micro Kit (Qiagen, Frederick, MD, USA). The SMART‐Seq v4 Ultra Low Input RNA Kit for Sequencing (Clontech, Hampshire, UK) was then used to prepare the cDNA libraries. Single‐end, 100 base‐pair read length sequencing was performed at the Lausanne Genomic Technology Facilities (Illumina HiSeq 2500 device). Quality checks were performed after the RNA extraction and after the cDNA library preparation using the Qubit and Fragment Analyzer and the Nextera XT DNA Library preparation kit (Illumina).

### 2.5 mRNA sequencing: data processing and statistical analysis

The raw sequencing reads were trimmed to remove the adapters and filtered for low quality and low complexity (Cutadapt v.1.3^14^ and seq_crumbs v.0.1.8 https://bioinf.comav.upv.es/seq_crumbs/). The reads were aligned (STAR v.2.4.2a)^15^ to the human genome (*Homo sapiens.GRCh38.82)* and the number of reads per gene locus was counted (htseq‐count v.0.6.1).^16^ The mRNA sequencing data has been deposited in the ArrayExpress database at EMBL‐EBI under accession number E‐MTAB‐8494 (https://www.ebi.ac.uk/arrayexpress/experiments/E-MTAB-8494).

Read count normalization, differential gene expression analysis and all subsequent analyses were performed using the statistical software R (v. 3.5.3). Genes expressed at a level of at least 1 count per million (cpm) in at least 1 sample were retained (*n* = 20485), and normalization factors were calculated using the trimmed mean of *M* values (TMM) method implemented in the edgeR package (v. 3.24.3).^17^ The counts data was subsequently transformed to log_2_ cpm and a linear model was fitted to each gene using the voom function implemented in the limma package (v. 3.38.3).^18^

Overall similarities among cell subsets were determined by performing a principal component analysis (PCA) using the top 500 most variable genes among all ILC and Th subsets.

Differential gene expression analysis was performed using the empirical Bayes statistics model for differential expression implemented in the limma package. First, we detected differentially expressed genes between total ILCs and total CD4 T cells. Second, we determined which genes were differentially expressed between each ILC subset and its mirror CD4 Th counterpart. Genes were considered as being differentially expressed in any comparison by using a *P*‐value threshold of 0.05 after Benjamini‐Hochberg *P*‐value adjustment.^19^

Gene set enrichment analysis (GSEA) was performed to determine enrichment of differentially expressed genes in gene ontology (GO) pathways (http://geneontology.org/) or custom gene lists related to chemokines and chemokine receptors, cytokines and cytokine receptors, checkpoint blockade, pattern‐recognition receptor (PRR) signaling, checkpoints, transcriptional regulation, and neuropeptide and neurotransmitter receptors.

GSEA was conducted similarly to the method described in Subramanian et al.^20^ for each ILC subset‐Th counterpart comparison separately. All genes detected by RNA sequencing were sorted after differential gene expression analysis according to their moderated *t*‐statistic estimate. For each gene list, an enrichment score (ES) was calculated for up‐ and down‐regulated genes separately by increasing or decreasing a running‐sum statistic proportionally to the magnitude of the *t*‐statistic of each gene (using *P *= 1 in equation 1 of Subramanian et al.).^20^ The genes included in the gene set were randomized 1000 times to obtain the normalized ES (NES) and associated *P*‐value The NES was calculated by dividing the ES by the mean of the randomized ES values, and the *P*‐value was equal to the proportion of randomized ES values that had a higher (for positive ES) or lower (for negative ES) value than the initial ES. The *P*‐values were then adjusted for the total number of gene sets tested by using the Benjamini‐Hochberg adjustment.

Heatmaps of row *z*‐scores were produced using the ComplexHeatmap package (v.1.20.0).^21^ For the spider charts, we defined a gene signature for each of the 6 cell subsets by selecting genes that were overexpressed in each cell subset compared to all other subsets. The number of genes included in each signature differed among subsets (ILC1 = 7, ILC2 = 55, ILCP = 593, Th1 = 2, Th2 = 6, and Th17 = 4). We subsequently calculated the average expression level of all genes within a signature in each cell subset independently, and drew the spider chart using the fmsb package (v.0.6.3).

### 2.6 RNA purification and qPCR

Total RNA was isolated from highly pure, sorted human ILC and CD4 Th cell subsets using the TRIZOL reagent according to the manufacturer's instructions (Invitrogen, Carlsbad, CA, USA). Final preparation of RNA was considered DNA‐ and protein‐free if the ratio of spectrophotometer (NanoDrop, ThermoFischer, Carlsbad, CA, USA) readings at 260/280 nm was ≥1.7. Isolated mRNA was reverse‐transcribed using the iScript cDNA Synthesis Kit (Bio‐RadLaboratories, Watford, UK) according to the manufacturer's protocol. The qPCR was carried out in the Applied Biosystems 7900HT Fast Real‐Time PCR Sequence Detection System (Applied Biosystems) with specific primers (hIL18R1 5′‐TGGTGTGGCAGTTAAGAGATG‐3′, 5′‐AGCACCAAGAGGTTGGATAAG‐3′; hIL1R1 5′‐CCTGCTATGATTTTCTCCCAATAAA‐3′, 5′‐AACACAAAAATATCACAGTCAGAGGTAGAC‐3′; hTLR4 5′‐AGTTGATCTACCAAGCCTTGAGT‐3′, 5′‐GCTGGTTGTCCCAAAATCACTTT‐3′; hST2 5′‐GAAAACCTAGTTACACCGTGGAT‐3′, 5′‐GCAAACACACGATTTCTTTCCTG‐3′; hPAX5 5′‐AAACCAAAGGTCGCCACAC‐3′, 5′‐GTTGATGGAACTGACGCTAGG‐3′; hTCF4 5′‐GGCTATGCAGGAATGTTGGG ‐3′, 5′‐GTTCATGTGGATGCAGGCTAC‐3′; hIL13RA1 5′‐TGAGTGTCTCTGTTGAAAACCTC‐3′, 5′‐GGGGTACTTCTATTGAACGACGA ‐3′; hVIPR1 5′‐TCATCCGAATCCTGCTTCAGA‐3′, 5′‐AGGCGAACATGATGTAGTGTACT‐3′; hVIPR2 5′‐CAGTGGCGTCTGGGACAAC‐3′, 5′‐CCGTCACTCGTACAGTTTTTGC‐3′; and hRAMP1 5′‐GGAGGCTAACTACGGTGCC‐3′, 5′‐CTCCCTGTAGCTCCTGATGG‐3′) using KAPA SYBR FAST qPCR Kits (KAPA Biosystems, Inc., MA). Samples were amplified simultaneously in triplicate in one‐assay run with a nontemplate control blank for each primer pair to control for contamination or for primer dimerization, and the Ct value for each experimental group was determined. The housekeeping gene (ribosomal protein S16) was used as an internal control to normalize the Ct values, using the 2^−ΔCt^ formula.

### 2.7 Statistical analysis

Statistical analysis was performed with GraphPad Prism software version 6 using parametric *t*‐test. The data is shown by plotting individual data points and the mean ± sem. A *P*‐value <0.05 (2‐tailed) was considered statistically significant and labelled with *. *P* values <0.01 were labelled with **.

## 3 RESULTS

### 3.1 Circulating human ILCs and CD4 Th cells have distinct gene signatures

To gain insight into the transcriptomic profiles of human ILC and CD4 Th cell subsets we isolated highly pure populations from peripheral blood of 3 healthy donors. Among total ILCs (Lineage^−^, CD127^+^), ILC subsets were purified according to published literature as CRTH2^−^cKit^−^CD56^−^ (ILC1s), CRTH2^+^cKit^+/−^ (ILC2s), and CRTH2^−^cKit^+^ (ILCPs). For CD4 Th cell subsets, chemokine receptors were used to distinguish CD4 Th1 (CXCR3^+^CCR6^−^), Th2 (CCR4^+^CRTH2^+^), and Th17 cells (CXCR3^−^CCR6^+^) among memory T cells (Supporting Information Fig. S1A). The expression of subset‐defining genes was verified in the subsequent mRNAseq analysis and enabled to confirm the high purity of the sorted populations, as well as the expression of subset‐defining master transcription factors (Supporting Information Fig. S1B‐C). By an initial comparison of the gene signatures of total ILCs and total CD4 Th cells, 1739 genes were found differentially expressed, 970 being up‐regulated in total ILCs as compared to total CD4 Th cells (Fig. 1A). As expected, *KIT* and *KLRB1* (encoding for CD161) were highly expressed in ILCs, whereas *CD4* and *TCR* related genes such as *TRAC*, *TRBC1*, and *TRBC2* were significantly higher in CD4 T cells than in ILCs. Other typical T cell genes, such as *CD2*, *CD3D*, and *CD3E* were present at low levels in ILC2 and in ILC3 but showed intermediate expression in ILC1, as compared to Th cells. In contrast, *TRDC* and *TRDJ2* were more expressed in ILCs than in CD4 Th cells, as previously reported by Li et al.^8^ (Supporting Information Fig. S1D).

Figure 1
**Differentially expressed genes between total innate lymphoid cells (ILCs) and total Th cells**. mRNA sequencing was performed on sorted ILC and Th subsets from the peripheral blood of 3 healthy donors. (**A**) Volcano plot showing the log_2_ fold change and significance of differential gene expression of genes in ILCs compared to Th cells. A total of 1739 genes (colored in green) were differentially expressed between total ILCs and total Th cells. (**B**) Venn diagram of shared gene ontology (GO) genesets that were significantly enriched in genes up‐regulated in ILCs compared to their respective Th counterparts (**C**) Heatmap (mRNAseq) of row *z*‐scores of the top 100 up‐regulated genes and the top 100 down‐regulated genes between total ILCs and total Th cells. Each gene is labeled with colors according to its belonging to the genesets listed. Genes were clustered hierarchically using the complete method on Euclidean distances of scaled log_2_ (normalized cpm)

**Figure:**
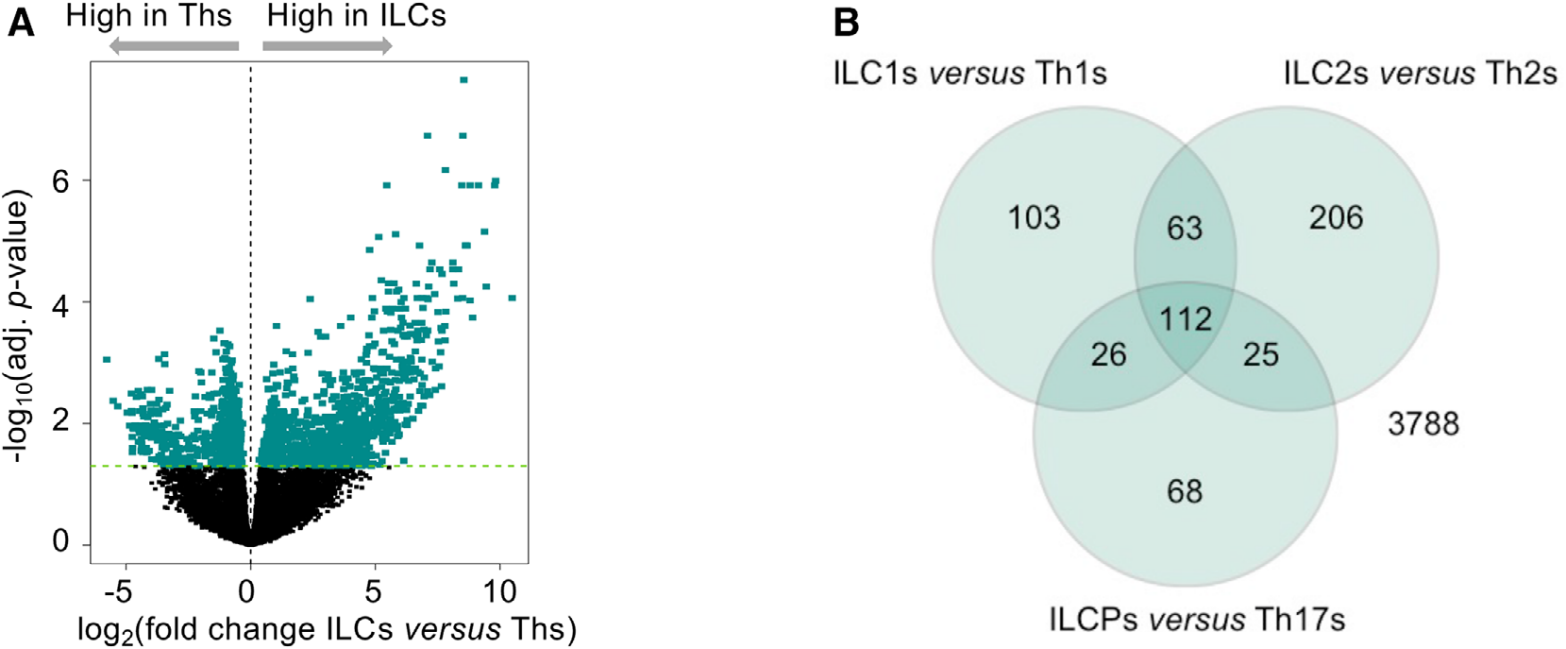

**Figure:**
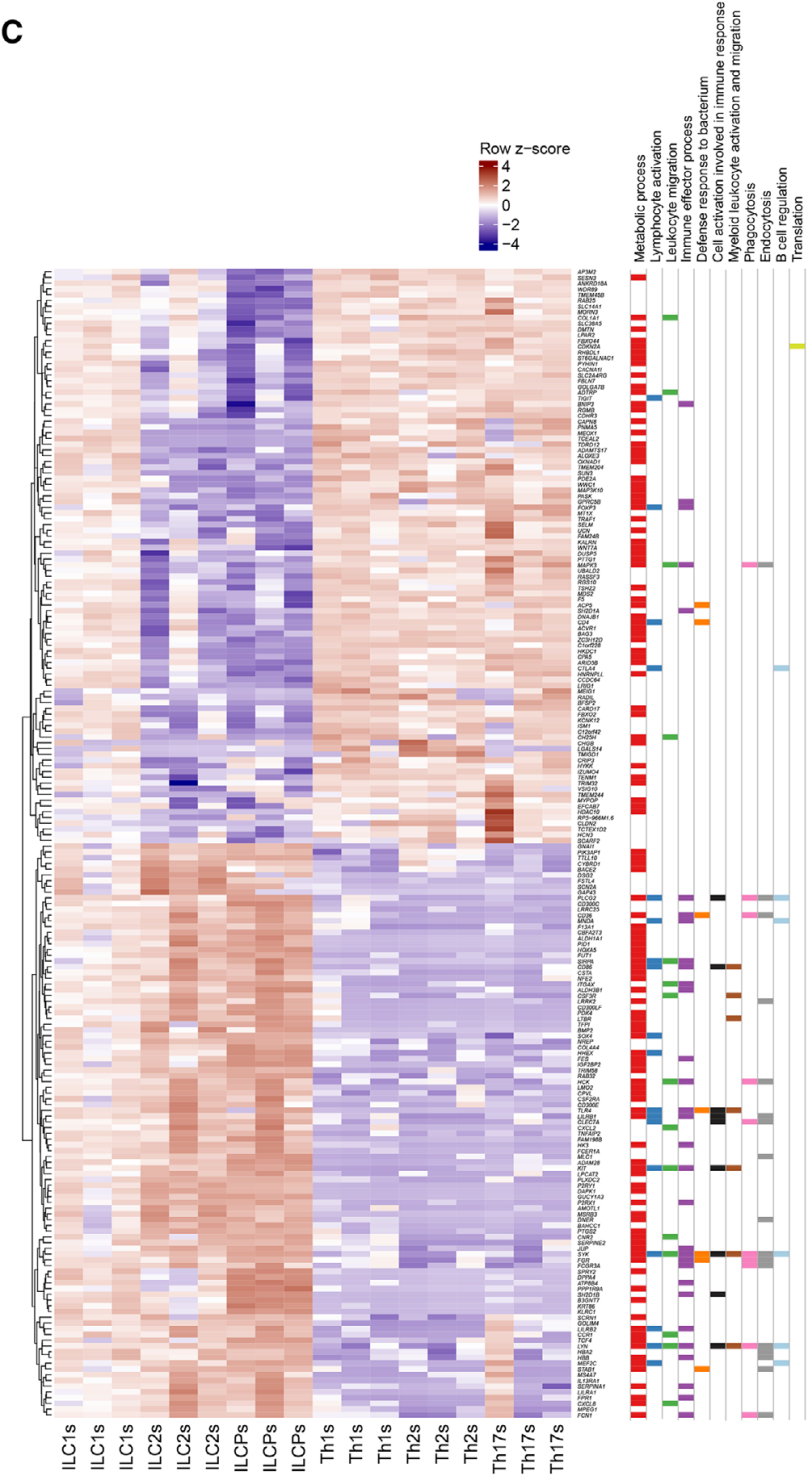

Next, we performed GSEA to determine whether defined GO genesets were shared as a common ILC signature as compared to the Th one. A total of 112 GO genesets were significantly enriched across all three individual ILC subsets vs. the Th subsets (Fig. 1B). These GO genesets related to metabolic processes, lymphocyte activation in immune responses, leukocyte migration, defense responses to pathogens, myeloid cell activation and migration, phago‐ and endocytosis, and B cell regulation (Fig. 1C). Because innate and adaptive effector cells interact with their surrounding environment through different types of receptors, we analyzed genes related to chemokines and cytokines, and their receptors, PRRs, activating and inhibitory coreceptors and receptors for neuropeptides. Among chemokines and chemokine receptors, we observed high expression of the chemokine receptor *CCR1* and of several chemokines including *CXCL2*, *XCL1*, and *PPBP* (an isoform of *CXCL7*) across ILC subsets in comparison to CD4 Th cells (Fig. 2A). High protein expression of *CCR1* on circulating ILCs was also confirmed by flow cytometry (Fig. 2A). Regarding cytokines, *LIF* and *IL18* were the most up‐regulated in ILCs, mainly due to high expression of these factors in ILCPs. The response of ILCs and CD4 Th cells to cytokines also seems to be distinctly regulated. Indeed, total ILCs expressed significantly higher levels of *IL13RA1*, *LTBR*, *IL18R1*, and *IL1R1*, as confirmed by qPCR or flow cytometry (Fig. 2B). The presence of these receptors in ILCs might account for the well‐known plasticity of these cells that are able to promptly convert one into the other in response to environmental cues. Moreover, given the absence of somatically rearranged antigen receptors on ILCs, these cells are characterized by their ability to rapidly respond to tissue mediators derived from lipid metabolism, bacterial, and dietary products. This is reflected in our mRNA sequencing data by the higher expression in ILCs of *TLR4*, involved in innate sensing of pathogens, and *CIITA*, the master regulator of MHC class II (Fig. 2C). Differences in the expression of master transcription factors might also account for the divergent differentiation of ILCs and Th cells. In that regard, we observed higher expression of *PAX5* and *TCF4* in ILCs than in Th cells (Fig. 2D).

Figure 2
**Flow cytometry or quantitative real time PCR (qPCR) validation of genes differentially expressed between total innate lymphoid cells (ILCs) and total Th cells**. All heatmaps show gene expression levels in the mRNAseq data. Gene symbols in heatmaps labeled with * are significantly differentially expressed between total ILCs and total Th cells. In qPCRs, the expression levels of genes were normalized to the expression of *RPS16*. (**A**) Heatmap of row *z*‐scores of chemokines and chemokine receptors, and flow cytometry validation of the higher expression of CCR1 at protein level in total ILCs compared to total Th cells. (**B**) Heatmap of row *z*‐scores of cytokines and cytokine receptors, and flow cytometry (protein level) or qPCR validation of the higher expression of *IL‐13RA1* (upper middle panel), *IL‐18R1* (upper right panel), *LTBR* (lower middle panels), and *IL‐1R1* (lower right panel) in total ILCs compared to total Th cells. (**C**) Heatmap of row z‐scores of TLRs and NOD‐like receptors, and qPCR validation of the higher expression level of *TLR4* in total ILCs compared to total Th cells. (**D**) Heatmap of row *z*‐scores of transcription factors, and qPCR validation of the higher expression level of *PAX5* and *TCF4* in total ILCs compared to total Th cells. (**E**) Heatmap of row *z*‐scores of inhibitory receptors, and flow cytometry validation of the lower expression of *CTLA4* and *TIGIT* at protein in total ILCs compared to total Th cells. (**F**) Heatmap of row *z*‐scores of adrenergic receptors, neuropeptide/neurotransmitter receptors, and glycolysis‐related genes. Data are shown as mean ± sem of three independent experiments (*P < 0.05)

**Figure:**
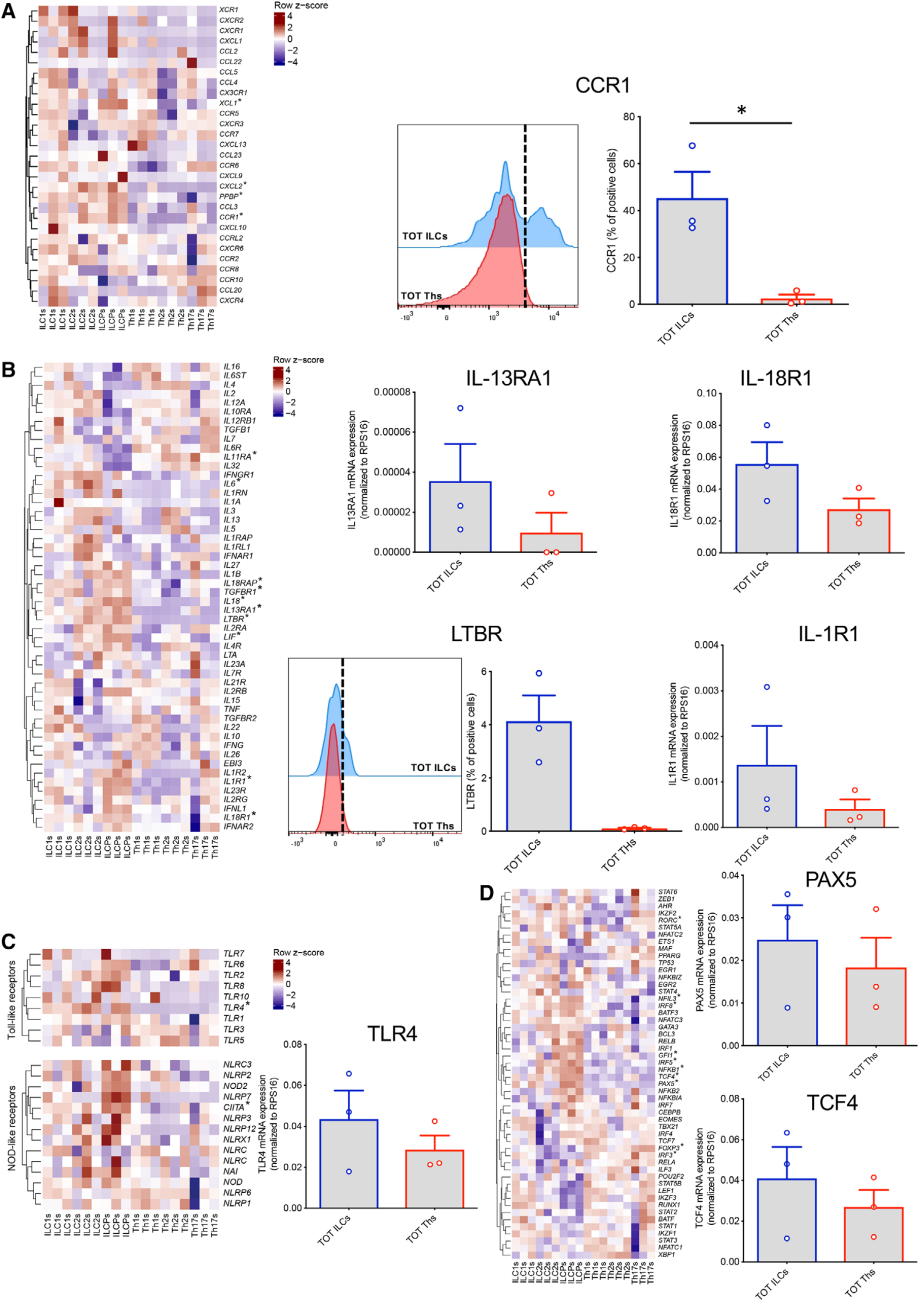

**Figure:**
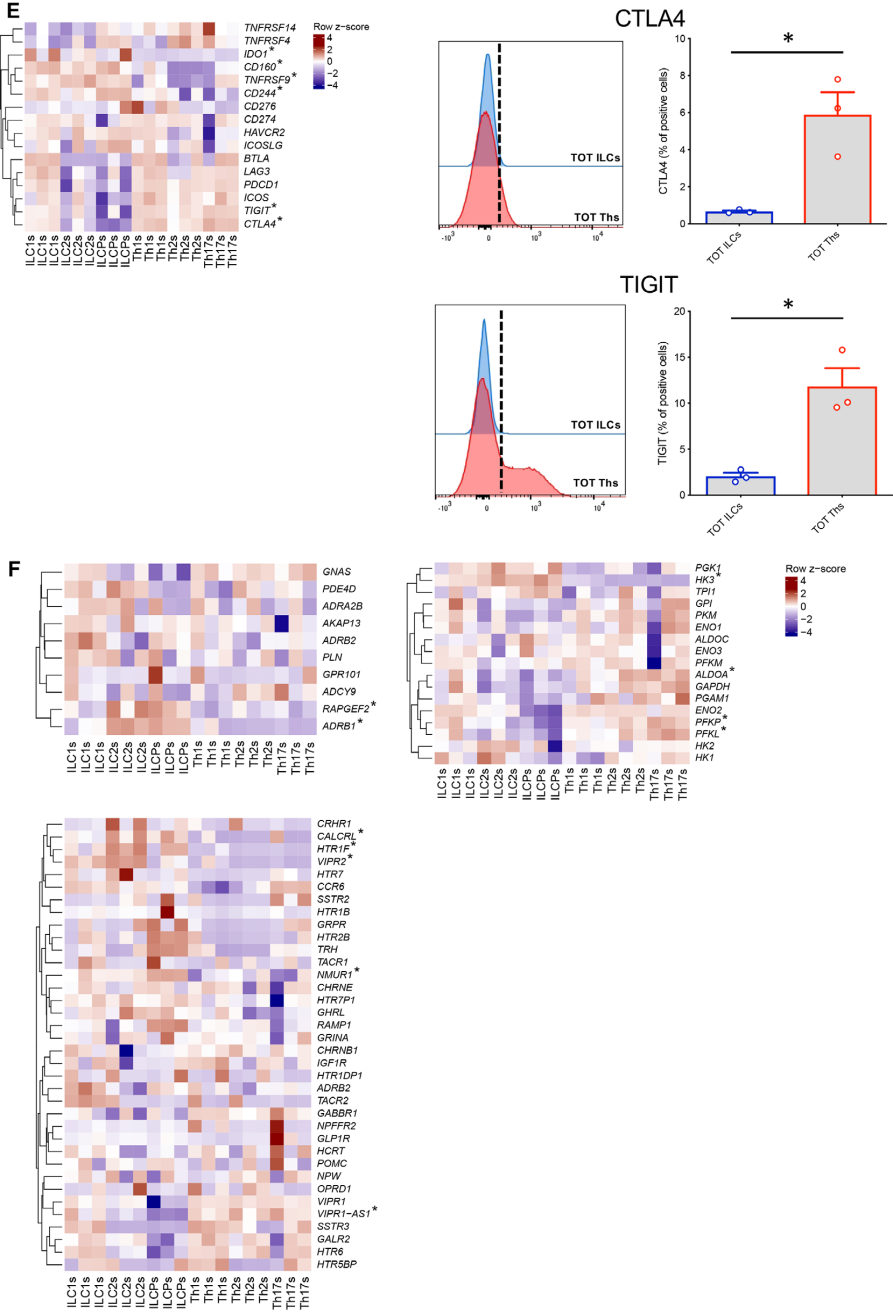

Finally, we compared the expression of costimulatory and coinhibitory receptors, and neuropeptide/adrenergic receptors known to influence the kinetics and magnitudes of immune cell responses. We identified a general absence or very low expression of inhibitory receptors (e.g., *TIGIT*, *CTLA4*) on total ILCs compared to CD4 Th cells, whereas only at mRNA level, higher expression of some costimulatory receptors, in particular of *4‐1BB* (*TNFRSF9*) (Fig. 2E). Moreover, in line with published work on ILC involvement in neural circuits,^22^, ^23^, ^24^, ^25^, ^26^ these cells showed overexpression of *ADRB1*, *VIPR2*, *NMUR1*, and *HTR1F* and significant differences in their metabolism‐related genes (Fig. 2F).

Overall, these results argue for distinct transcriptomic signatures in human ILCs and CD4 Th cells that might be related to their distinct sensing of the environment and kinetics of immune reactivity.

### 3.2 Pairwise comparison of mirror ILCs and CD4 Th cell subsets reveals shared, but also distinct gene profiles

**Figure 3:**
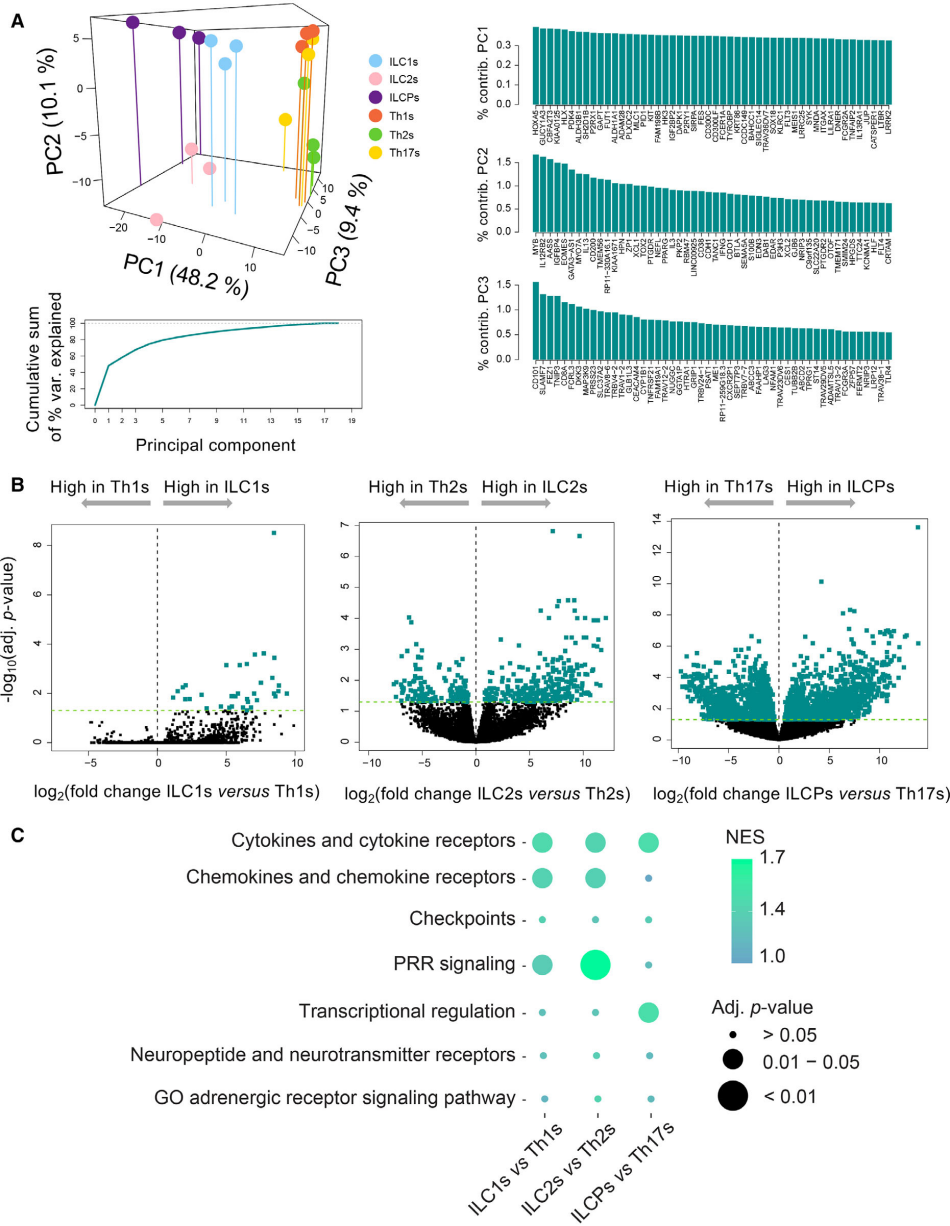
**Distinct transcriptional profiles between each innate lymphoid cell (ILC) subset and their respective Th counterparts**. (**A**) Three‐dimensional plot of a principal component analysis of 3 ILC subsets and their three Th counterparts, cumulative sum of percentage variance explained by 18 principal components (PCs), and percentage contribution of the top 50 genes to the three first PCs. (**B**) Volcano plot showing the log_2_ fold change and significance of differential gene expression of genes in each ILC subset and its respective Th counterpart. Differentially expressed genes are colored in green. (**C**) Geneset enrichment analysis of genesets related to chemokines/chemokine receptors, cytokines/cytokine receptors, immune inhibitory proteins, pattern recognition receptors, transcriptional regulation, and neuropeptide/neurotransmitter receptors. The enrichment in these genesets was tested using genes up‐regulated between each ILC subset and its Th counterpart. The color scale from blue to green shows the magnitude of the normalized enrichment score (NES), and the size of each dot is proportional to the adjusted *P*‐value associated to each NES

Next, we aimed at directly comparing the gene expression profiles of mirror ILCs and CD4 Th cell subsets. To do that, we performed a PCA of ILC1s, ILC2s, ILCPs, Th1s, Th2s, and Th17s using the top 500 most variable genes among ILCs and CD4 Th subsets (Fig. 3A, B). Forty‐eight percentage of the variability was explained with the first principal component (PC), which represents the dichotomies of ILCs vs. Th cells and ILCPs/ILC2s vs. ILC1s. Interestingly, ILC1s, ILC2s and ILCPs were clearly separated from each other along the first PC. However, the 67.7% of variance explained by the three first PCs did not allow to clearly distinguish the Th subsets among each other, in particular when comparing Th1 and Th17 cells. Further, by performing pairwise differential gene expression analysis of ILC1s and Th1s, ILC2s and Th2s and ILCPs and Th17s a high number of genes was found to be differentially expressed, as reported in the volcano plots of Figure 3B and summarized in Table 1. The most striking difference in transcript expression was observed in the comparison of ILCPs and Th17 cells, with a total of 3192 differentially expressed genes, 1566 of them being overexpressed in ILCPs. Between ILC2s and Th2 cells, 442 genes were differentially expressed, 281 of these being more expressed in ILC2s. Finally, only 38 genes were significantly differentially expressed between ILC1s and Th1 CD4 T cells, probably due to the known heterogeneity of the ILC1 subset, for which no specific surface marker has been defined yet. To better understand which genes or pathways accounted for the differences between ILC subsets and their respective CD4 Th counterparts we further explored the individual contribution of GO genesets to the distinction between paired subsets (Fig. 3C).

**Table 1:** Number of significantly differentially expressed genes among innate lymphoid cell (ILC) and Th subsets

Up‐regulated genes						
	ILC1s	ILC2s	ILCPs	Th1s	Th2s	Th17s
**ILC1s**		13	396	0	5	2
**ILC2s**	65		387	369	161	640
**ILCPs**	738	320		1363	1227	1626
**Th1s**	38	400	1065		41	9
**Th2s**	71	281	1151	19		14
**Th17s**	28	549	1566	1	20	
Down‐regulated genes

	ILC1s	ILC2s	ILCPs	Th1s	Th2s	Th17s
**ILC1s**		65	738	38	71	28
**ILC2s**	13		320	400	281	549
**ILCPs**	396	387		1065	1151	1566
**Th1s**	0	369	1363		19	1
**Th2s**	5	161	1227	41		20
**Th17s**	2	640	1626	9	14	

Each table is read column‐wise. For example, the first column in the first table indicates the genes that are up‐regulated in ILC1s as compared to the other subsets indicated in rows, whereas the first column in the second table indicates the genes that are down‐regulated in ILC1s compared to the other subsets indicated in rows.

### 3.3 GSEA of differentially expressed genes between mirror subsets

**Figure 4:**
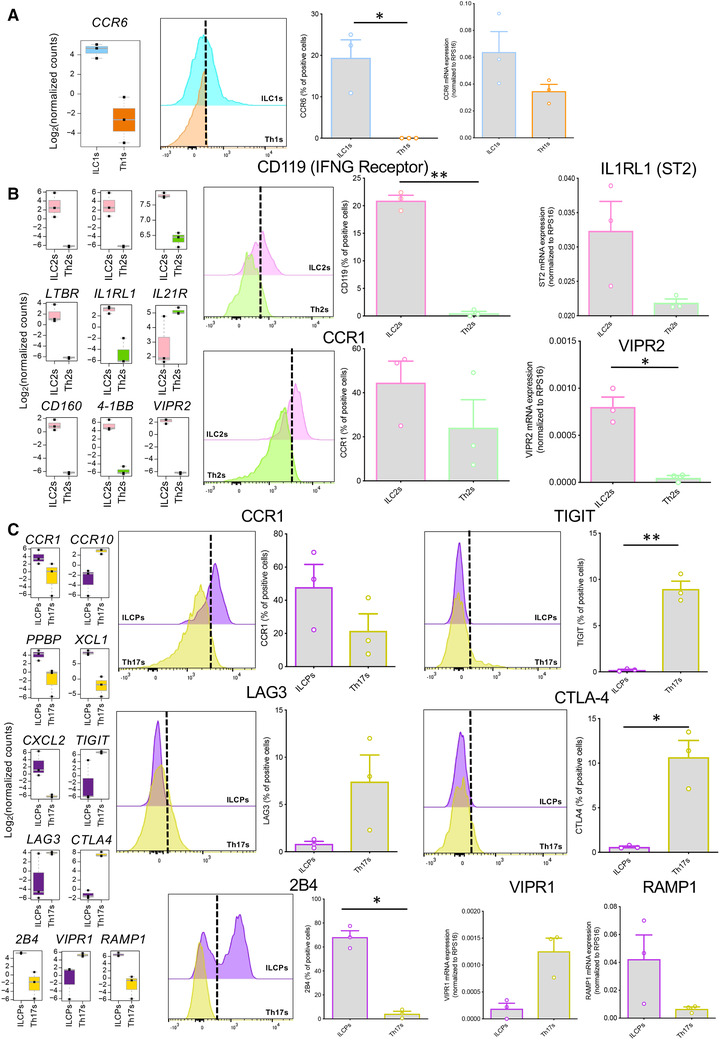
**Flow cytometry or qPCR validation of genes differentially expressed between each innate lymphoid cell (ILC) subset and its respective Th counterpart**. All boxplots show the gene expression level in the mRNAseq data. In qPCRs, the expression levels of genes were normalized to the expression of *RPS16*. (**A**) Boxplot (left panel) and qPCR validation (right panel) of the higher expression of *CCR6* in ILC1s compared to Th1s. (**B**) Boxplots and flow cytometry or qPCR validation of the higher expression of *IFNGR1* (upper mid panels), *IL1RL1* (upper right panel), *CCR1* (lower mid panels) and *VIPR2* (lower right panel) in ILC2s compared to Th2s. (**C**) Boxplots and flow cytometry or qPCR validation of differential expression of *CCR1* (upper mid panels), *TIGIT* (upper right panels), *LAG3* (central mid panels), *CTLA4* (central right panels), *2B4* (lower mid panels), *VIPR1* (lower right panel), and *RAMP1* (lower right panel) between ILCPs and Th17s. Data are shown as mean ± sem of three independent experiments (*P < 0.05; **P < 0.01)

Whereas Th1s are defined as CCR6^−^ cells, we found a significant higher expression of *CCR6* in ILC1s compared to Th1s at both transcriptomic and protein level (Fig. 4A). ILC2s were characterized by high mRNA levels of *CCR1* and *CXCL2*, *IFNGR1* (also known as CD119), *LTBR*, *IL1RL1* (also known as *ST2*, the IL‐33 receptor), *CD160*, *4‐1BB*, and significantly lower expression of the *IL21R*, as compared to their Th counterparts (Fig. 4B). Elevated protein levels of CCR1 and IFNGR1 were confirmed by flow cytometry and increased transcript for *ST2* were validated by qPCR (Fig. 4B). Expression of *VIPR2* was also higher in ILC2s (Fig. 4B). Finally, in the comparison between ILCPs and Th17 cells the most striking differences appeared in CCR1 expression, that was higher in ILCPs, and *CCR10* that was increased in Th17 cells. Several chemokines were also overexpressed in ILCPs, including XCL1, PPBP, and CXCL2. Furthermore, when considering costimulatory and coinhibitory receptors, Th17 cells presented elevated levels of TIGIT, LAG3, and CTLA4, whereas ILCPs were overexpressing 2B4, as confirmed at protein level. Neuroregulatory circuits might also be distinct among ILCPs and Th17s, as illustrated by the different expression of *VIP1R* and *RAMP1* (Fig. 4C).

**Figure 5:**
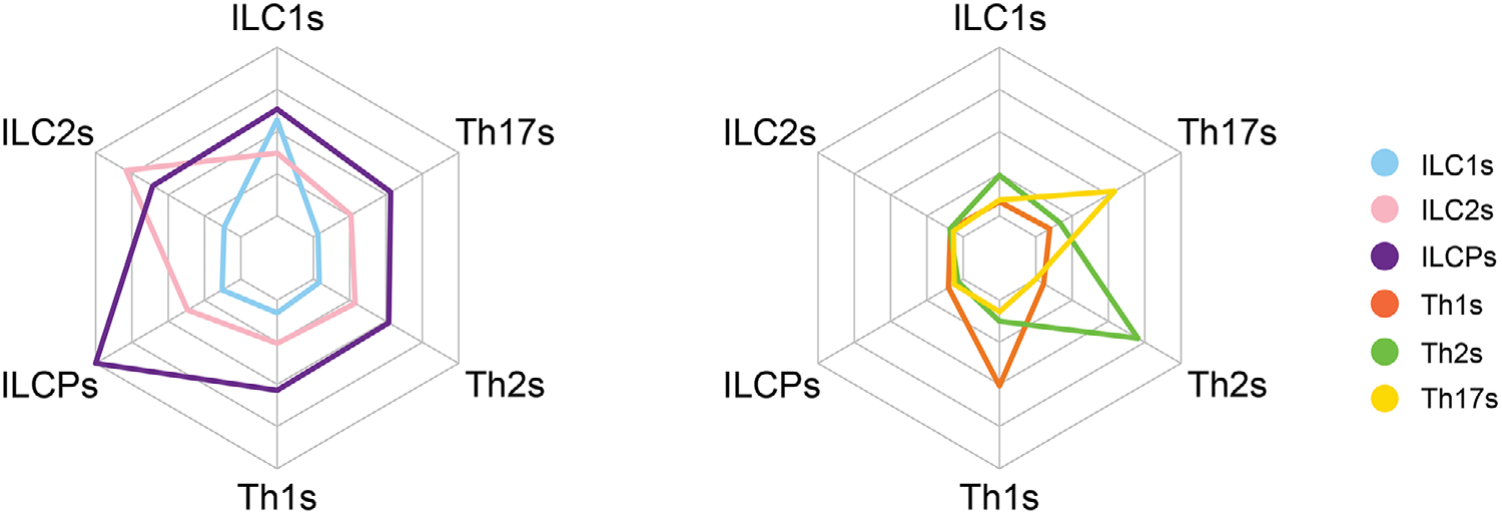
**Each innate lymphoid cell (ILC) or Th subset overexpresses a different set of genes**. For each cell subset, we defined a gene signature that consisted of the genes up‐regulated in each subset compared to all 5 other subsets. The 6 tips of the net in both charts represent the 6 individual gene signatures specific to each cell subset. The colored lines in each chart show the average expression level of the genes within each of the 6 signatures, for ILCs (left panel) and Th cells (right panel) separately. The scale of the average expression level (unit: log_2_ [normalized cpm]) from the center of each chart to the outer line ranges from −6.2 to +3.5. Genes specifically overexpressed in ILC2s or ILCPs are expressed at high level within these two subsets but are expressed at intermediate levels within ILC1s and the Th cells. On the other hand, genes specifically overexpressed in ILC1s, Th1s, Th2s, or Th17s are expressed at intermediate levels within each respective subset but are very lowly expressed in other subsets

Overall, to visualize the contribution of these genes in defining the ILC subset signature as compared to their CD4 Th counterparts a spider diagram representation was used. As shown in Figure 5, ILCPs and Th2 cells have the most conserved prototypical signature among the tested cell subsets, as well as the highest number of genes overexpressed compared to all other subsets. ILCPs, and to a lesser extent ILC2s, overexpress some genes present across all the different ILC and Th subsets. Similar to ILC1s, Th subsets overexpress genes that are absent in the other subsets, and appear to be highly committed toward their given gene signature.

### 3.4 Expression of lncRNAs is distinct between ILCs and CD4 Th cells

**Figure 6:**
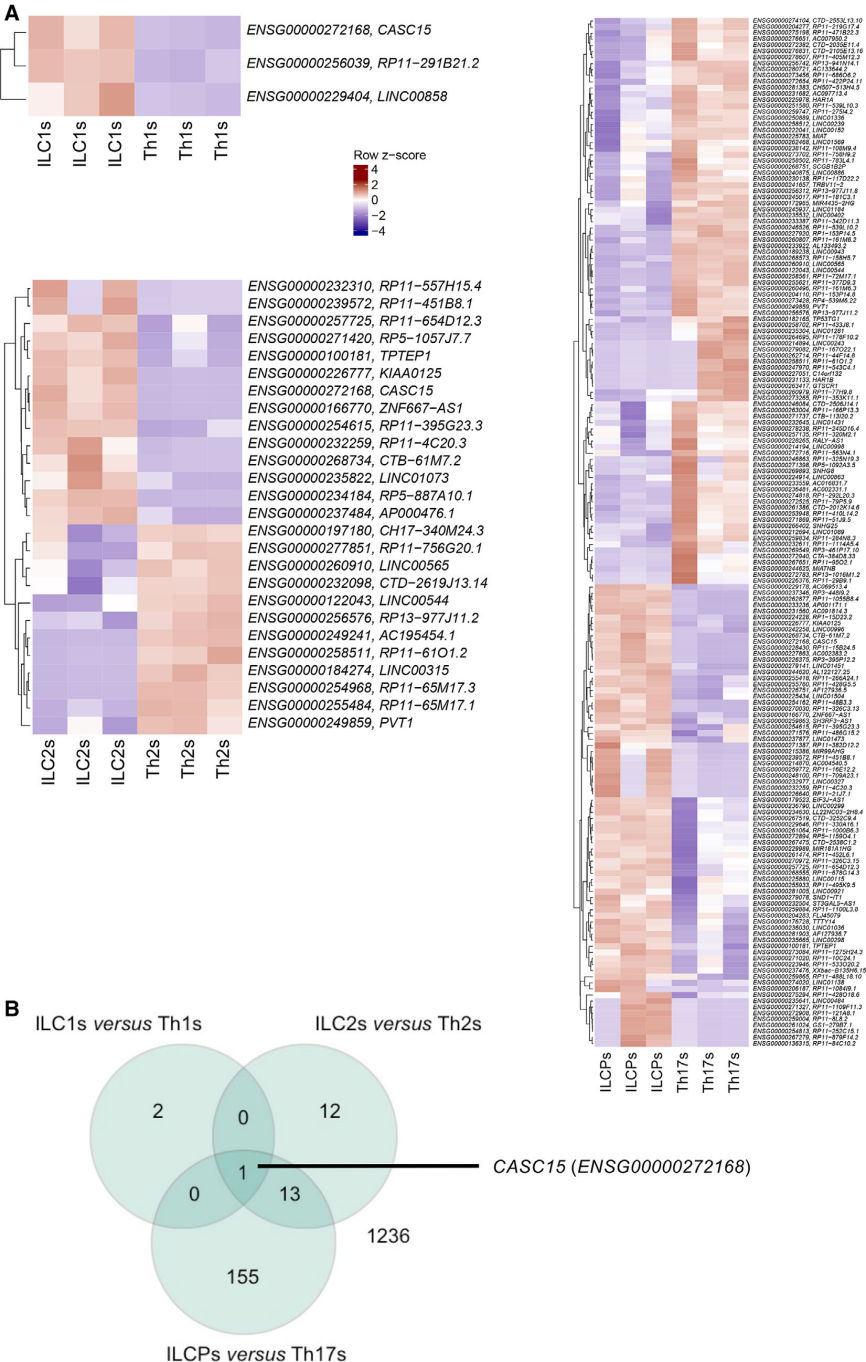
**Distinct transcriptional profiles of long noncoding RNAs in each innate lymphoid cell (ILC) subset and its Th counterpart**. (**A**) Heatmap (mRNAseq) of row *z*‐scores of all lncRNAs differentially expressed between ILC1s and Th1s, ILC2s and Th2s, and ILCPs and Th17s

The lncRNAs represent an abundant part of the cellular mRNA content, display high cell subtype specificity, and their expression has been associated with several diseases.^27^ Therefore, we explored our dataset for the expression levels of lncRNAs in ILCs as opposed to CD4 Th cells. A sharp differential expression of lncRNAs between the innate and adaptive counterparts could be observed, with 3, 26, and 169 lncRNAs differentially expressed between ILC1s/Th1s, ILC2s/Th2s, and ILCPs/Th17s, respectively (Fig. 6A). Interestingly, a shared expression of lncRNAs was observed among ILC subsets, as represented in the Venn diagrams of Figure 6B. One lncRNA in particular, CASC15, was overexpressed by ILCs regardless of specific subset, as opposed to their Th counterparts and it might represent an ILC‐specific lncRNA signature gene.

## 4 DISCUSSION

Here, we provide for the first time a comprehensive transcriptomic comparison between human circulating ILC and CD4 Th subsets. Our results argue for complementary rather than redundant functions of these cell populations, likely reflecting the different manner of activation and the distinct kinetics of responses of these innate and adaptive helper counterparts.

The expression patterns of chemokine and cytokine receptors clearly differ among ILCs and Th cells is in their chemokine and cytokine receptor expression. In particular, *CCR1*, the receptor for MIP‐1 alpha, RANTES and CCL23, and *CCR6*, the receptor for CCL20, were found in blood ILC subsets at steady state. Chemokine‐chemokine receptor interactions are well‐known drivers of immune cell migration to tissues. Therefore, the observation that human circulating ILCs are equipped with distinct patterns of chemokine receptors supports the view that ILCs are not exclusively tissue resident cells reacting only to tissue signals, where they are localized.^28^ The different expression of these receptors might guide ILCs into different tissues as compared to Th cells, a process that might be key in their deployment during early stages of inflammation and infections. ILCs also showed increased levels of expression of cytokine receptors, such as *IFNGR*, *IL1R*, *IL13R*, *IL18R*, and *LTBR*. Interestingly, each subset is expressing cytokine receptors not primarily involved in that given subset specification/maintenance. It is tempting to speculate that the presence of these receptors accounts for a preprogrammed subset plasticity enabling the rapid adaptation in effector functions in the microenvironment (e.g., *IFNGR* on ILC2s). In this regard, evidence of ILC plasticity has been extensively reported in ILC1s,^29^ ILC2s,^30^, ^31^, ^32^, ^33^ and ILC3s.^34^ To some extent, plasticity among Th subsets has also been described, mainly between Th1/Th2^35^, ^36^ and Th17/regulatory T cells (Tregs).^37^

Further, the observed overall low levels of coinhibitory receptors on ILCs agree with a cell profile characteristic of more immediate activation, to provide protection in a window when CD4 Th cells have not yet developed a specific immune response. However, expression of *PD‐1* has been reported in ILC2s,^38^ where it acts as STAT5‐dependent negative regulator of ILC2 functions. Our observations suggest that at steady‐state, in the circulation, human ILCs are poorly equipped with immune checkpoints, whereas they might acquire them in tissues and/or under inflammatory conditions, as recently reported for NK cells.^39^, ^40^

In the absence of rearranged antigen receptors, ILCs display a response pattern that is restricted to a limited array of stimuli. Of interest, beside the expected increased expression of PRRs as compared to Th subsets, ILCs appear to also be sensitive to nontypical pathogen associated patterns, such as neurotransmitters. Evidence for neuroendocrine regulation of ILCs has recently been proposed. For instance, Quatrini et al. reported that glucocorticoids affect ILC1 functions upon murine cytomegalovirus (MCMV) infection in a spatial and cell‐specific manner.^41^ Further, neural circuits in the respiratory and intestinal tracts were shown to modulate ILC2 and ILC3 functions by interacting with CALCRL,^42^ VIPR1/2,^25^ and NMUR1^21,22,24^ expressed on these cells. How this regulation impacts human physiologic and pathologic processes remains to be fully addressed. *CALCRL* and *VIPR2* gene expressions are higher in ILC2s compared to Th2s, suggesting that ILC2s might be more susceptible to neural‐induced signals than Th2 cells.

Fine‐tuning of immune cell functions and acquisition of cell identity might also result from the effect of lncRNAs on gene expression. In CD4 T cells, LincR‐Ccr2‐5′AS has been involved in murine CD4 Th2 cell migration^43^ and linc‐MAF‐4 was described as specifically linked to a CD4 Th1 phenotype.^44^ With respect to ILCs, a previous work identified the lncRNA *Rroid* as a key regulator of ILC1 development in mice.^45^ In our analysis we observed significantly different expression of lncRNAs in ILCs as compared to CD4 Th cells. This is particularly true for the ILCPs vs. Th17s comparison, where more than 150 lncRNAs were found to be differentially expressed. ILCPs are known to maintain open developmental options toward both NKs and helper ILCs.^46^ It might be speculated that this innate cell precursor abilities are at least in part controlled by the action of lncRNAs and other epigenetic cues, as recently shown in ILC2 development.^47^ Future studies will be needed to evaluate the impact of distinct lncRNAs on ILCs. Of interest, we identified CASC15 as a signature lncRNA in ILCs as opposed to total CD4 Th cells. Several studies have already evaluated the role of this lncRNA in different types of tumor. In that regard, CASC15 has been reported to directly bind to the enhancer of zeste homolog 2 (EZH2), the key catalytic component of the polycomb repressive complex 2 (PRC2) involved in H3‐K27 methylation.^48^ In melanoma, the CASC15‐dependent EZH2‐mediated inhibition of PDCD4 resulted in tumor progression.^49^ Whether this lncRNA has also an impact on ILC development and/or proliferation remains to be investigated.

Overall, our transcriptomic results support the notion that ILCs might be involved in physiologic process or contribute to pathologies previously attributed to T cell functions/dysfunction.^50^ We also provide indication for potential hitherto unknown roles of lncRNA in shaping human ILC biology. Final evidence in support of this assumption will need investigations in mouse models specifically probing gene expression in ILC subsets.

## AUTHOR CONTRIBUTIONS

GE performed the experiments, analyzed the data and wrote the paper. TW performed bioinformatics analysis. BS performed the experiments, provide intellectual contributions and revised the manuscript. PR provided intellectual contribution and revised the manuscript. ST provided intellectual contribution, supervised the flow cytometric analysis and revised the manuscript. CJ provided intellectual contribution, supervised all the experiments, critically revised the manuscript and gave final approval to the publication.

## DISCLOSURES

The authors declare no conflicts of interest.

## AUTHORSHIP

E.G. and W.T. contributed equally.

## Supporting information

Supporting Information
