# Supplementary figures and images for: Distinct and shared gene expression for human innate versus adaptive helper lymphoid cells
Source: J Leukoc Biol. 2020 Feb 4;108(2):723–37. doi: 10.1002/JLB.5MA0120-209R (PMC7496918; doi:10.1002/JLB.5MA0120-209R)

Figure S1

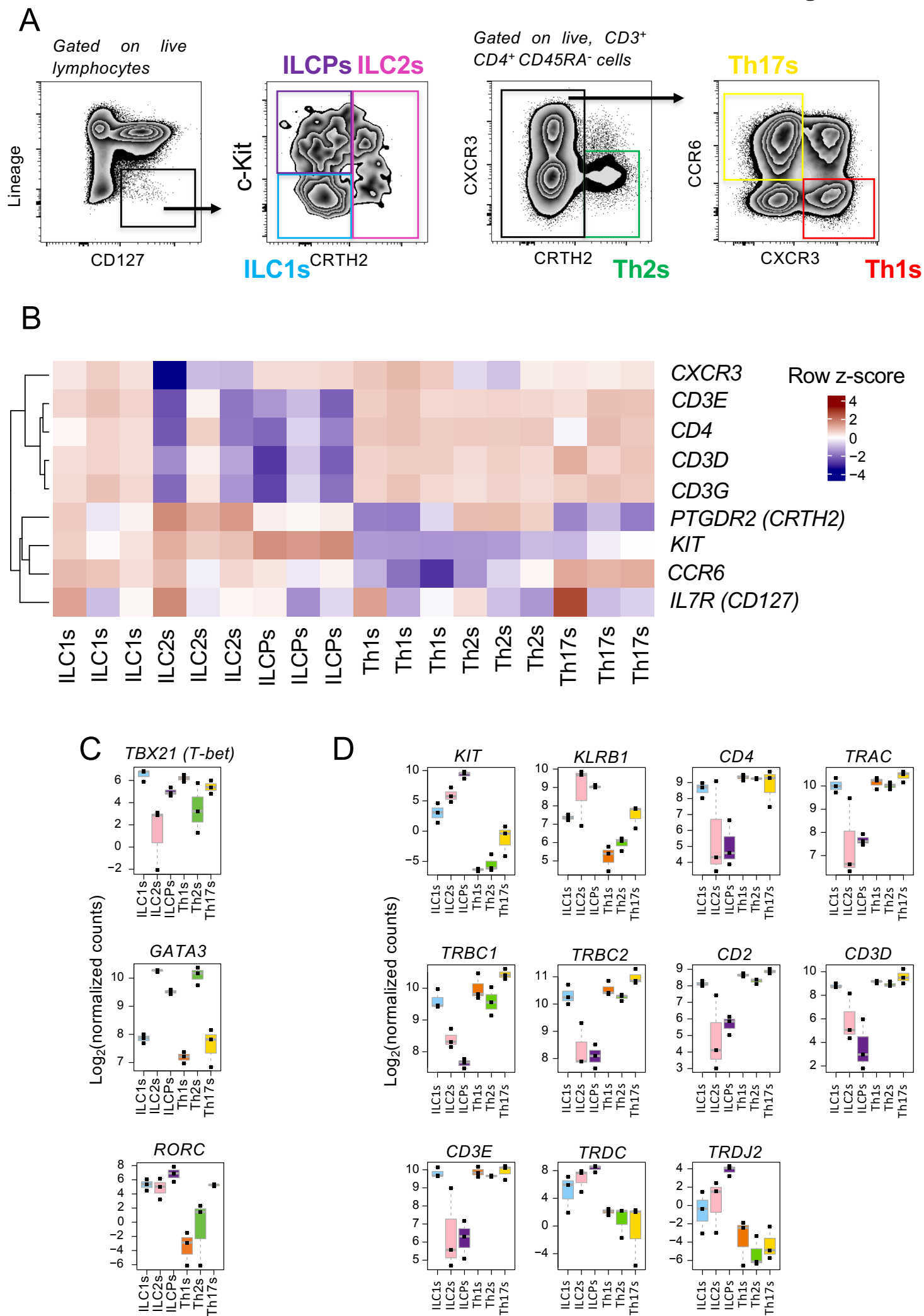

Supplement: Supplementary file 1 — Supporting Information [file JLB-108-723-s001.pdf]
